# Homeostasis Maintenance in *Plasmodium*-Infected Placentas: Is There a Role for Placental Autophagy During Malaria in Pregnancy?

**DOI:** 10.3389/fimmu.2022.931034

**Published:** 2022-07-11

**Authors:** André Barateiro, Alexsander Rodrigues Carvalho Junior, Sabrina Epiphanio, Claudio Romero Farias Marinho

**Affiliations:** ^1^ Institute of Biomedical Sciences, Department of Parasitology, University of São Paulo, São Paulo, Brazil; ^2^ School of Pharmaceutical Sciences, Department of Clinical and Toxicological Analysis, University of São Paulo, São Paulo, Brazil

**Keywords:** Malaria, *Plasmodium*, Pregnancy, Placenta, Inflammation, Autophagy

## Abstract

Malaria represents a significant public health burden to populations living in developing countries. The disease takes a relevant toll on pregnant women, who are more prone to developing severe clinical manifestations. Inflammation triggered in response to *P. falciparum* sequestration inside the placenta leads to physiological and structural changes in the organ, reflecting locally disrupted homeostasis. Altogether, these events have been associated with poor gestational outcomes, such as intrauterine growth restriction and premature delivery, contributing to the parturition of thousands of African children with low birth weight. Despite significant advances in the field, the molecular mechanisms that govern these outcomes are still poorly understood. Herein, we discuss the idea of how some housekeeping molecular mechanisms, such as those related to autophagy, might be intertwined with the outcomes of malaria in pregnancy. We contextualize previous findings suggesting that placental autophagy is dysregulated in *P. falciparum*-infected pregnant women with complementary research describing the importance of autophagy in healthy pregnancies. Since the functional role of autophagy in pregnancy outcomes is still unclear, we hypothesize that autophagy might be essential for circumventing inflammation-induced stress in the placenta, acting as a cytoprotective mechanism that attempts to ensure local homeostasis and better gestational prognosis in women with malaria in pregnancy.

## Poor Pregnancy Outcomes due to Dysregulation of Placental Homeostasis in Women With Malaria in Pregnancy

Malaria represents a burden for multiple communities worldwide. Despite continuous eradication efforts, 241 million cases were recorded in 2020, resulting in approximately 627 thousand deaths. The disease affects mostly African children and pregnant women, who are highly susceptible to developing severe disease resulting from *P. falciparum* infections (1). Malaria in pregnancy (MiP) is often neglected, yet in 2019, the World Health Organization raised awareness of this issue, describing MiP as “a significant public health problem”. In 2020, the disease affected nearly 12 million African women who gave birth to more than 800 thousand babies with low birth weight, contributing to high infant mortality (1, 2).

*P. falciparum* infections during pregnancy often lead to poor outcomes, such as maternal and fetal mortality, anemia, fetal growth restriction, preterm delivery, and low birth weight (3, 4). Disease susceptibility varies according to *P. falciparum* endemicity spectrum, as pregnant women living in high transmission settings tend to have symptomless infections due to recurrent exposure during pregnancy. Nevertheless, women in their first pregnancy may experience more severe symptoms due to low immunity (5). Immunity is governed by protective antibodies to VAR2CSA (6, 7), which mediates the binding of *P. falciparum*-infected erythrocytes to chondroitin sulfate A, abundantly expressed by trophoblasts in the placenta (8, 9). This event promotes parasite sequestration inside this organ, which characterizes placental malaria (PM) (10). Trophoblasts (and eventually maternal monocytes) in the placenta recognize *P. falciparum* antigens, activating innate immune responses, which culminate with the production of chemokines such as MIP-1α, MCP-1, I-309, and IL-8 (responsible for monocyte recruitment) and cytokines, such as IFN-γ, TNF-α, IL-2, IL-6, IL-10, and IL-1β, which are frequently found in placentas from *P. falciparum-*infected women (11–15). Inflammation is known to control infection, yet it is etiologically linked to poor outcomes (16, 17) since both placental monocytes and cytokines have been associated with maternal anemia, pregnancy loss, preterm delivery, and low birth weight in children born to *P. falciparum*-infected women (13, 14, 18–20).

The placenta ensures immunological tolerance to the maternal immune system (21), promotes nutrient and gas exchanges between the mother and the fetus (22), and works as a physical and immunological barrier against pathogens (23). These properties are lost during infections due to significant tissue disarrangement. *P. falciparum*-infected placentas often suffer from histological modifications, such as the presence of malarial pigment hemozoin, leukocyte infiltrate, syncytial nuclear aggregates, fibrinoid necrosis, and trophoblast barrier thickening (24–26). Therefore, it is possible that this reflects local homeostasis dysregulation, which would predict fetal growth restrictions due to placental insufficiency resulting from impaired placental blood flow and vasculogenesis/angiogenesis, hypoxia, oxidative stress, and reduced transplacental nutrient transportation (3, 27).

Although several factors are known to be associated with MiP outcomes, the molecular mechanisms involved in disease pathogenesis are still unclear. Therefore, it is possible that other mechanisms less studied to date, such as those involving autophagy, might be linked to MiP pathogenesis.

## Autophagy

### Regulation and Function in Homeostatic and Stressful Conditions

**Figure 1:**
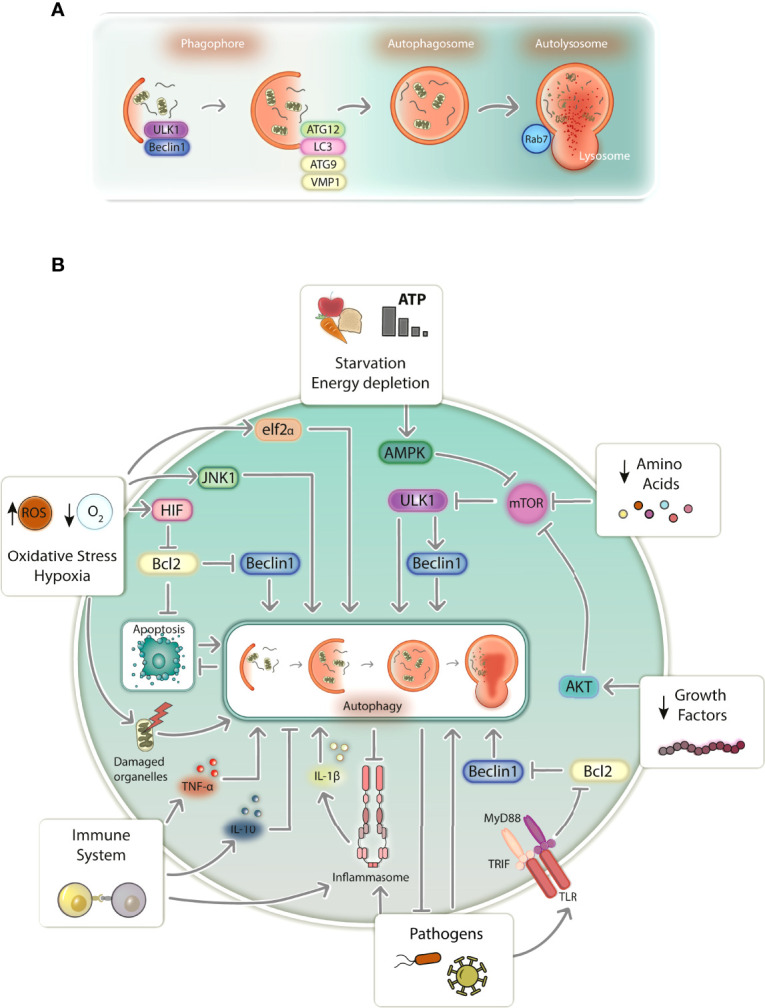
Schematic representation of autophagy in mammalian cells. **(A)** Autophagy proceeds through a series of events that begin with phagophore formation, elongation, and maturation into an autophagosome. The autophagosome fuses with a lysosome to form the autolysosome, followed by the degradation of selected cargo. Autophagy is tightly regulated by protein complexes produced from autophagy-related genes (ATGs) and its execution relies mostly on ULK1 and Beclin1 complexes (phagophore isolation), ATG9 and VMP1 (phagophore elongation), the ATG12 complex and LC3 (phagophore maturation into autophagosomes), and Rab7 GTPase (autophagosome-lysosome fusion for autophagy completion). **(B)** Cells transduce distinct signals that equally induce autophagy. Starvation, growth factor and amino acid depletion induces autophagy *via* AMPK and AKT, which blocks the inhibitory action of mTOR over the autophagy inducers ULK1 and Beclin1. Oxidative stress and hypoxia induce autophagy *via* elf2α, JNK1 and HIF, being the latter responsible for disrupting the inhibitory action of Bcl2 over Beclin1. Damaged organelles such as mitochondria induce autophagy, which digests damaged organelles and prevents cells death resulting from apoptosis. Immune system activation induces autophagy *via* TLR signaling upon pathogen sensing by destabilizing Bcl2-Beclin1 interaction. Autophagy is also activated or inhibited by TNF-α and IL-10, respectively. Inflammasome activity also induces autophagy, which works to counteract its overactivation resulting from excessive inflammatory signals. Pathogens directly induce autophagy, besides activating the immune system, which suppresses pathogen development by directly targeting them for degradation. ROS, reactive oxygen species; TLR, Toll-like receptor.

Autophagy is conventionally known as a conserved mechanism of lysosomal degradation of cytoplasmic components and is involved in cellular differentiation and development (28), homeostasis and survival (29). It can be activated in response to a plethora of stress-inducing agents that range from starvation, hypoxia, oxidative stress and damaged organelles to immune activation and pathogens, promoting degradation of undesired microorganisms, nutrient recycling, and organelle turnover (29–31) (**Figure 1**). In mammalian cells, the most well studied type of autophagy is macroautophagy (hereafter referred to simply as autophagy), which relies on the *de novo* formation of a vesicular structures capable of selecting and transporting cargo to lysosomes for degradation (32). Proteins transcribed by autophagy-related genes (ATGs) tightly regulate this process, which is primarily characterized by the formation of an intermediate double-membrane structure (phagophore) that surrounds specific cargo (forming the autophagosome), ultimately fusing with lysosomes (autolysosomes), which promote content digestion (29). The system seems to be redundant, as different triggers converge to activate almost identical transduction pathways inside cells (31). At its core, autophagy is regulated by at least some primary core complexes (**Figure 1A**): 1) the ULK complex (2), the Beclin1 interactome, 3) ATG9 and VMP1 transmembrane proteins, 4) two ubiquitin-like conjugation systems associated with ATG12 and LC3, and 5) autophagosome-lysosome fusion mediators (i.e., Rab7). Some of these components can be directly activated by distinct stress-inducing signals, likely ending with autophagosome-lysosome fusion and digestion of inner-vesicle cargo (29).

Several molecules can induce autophagy in response to starvation (**Figure 1B**); however, the best studied are those associated with mTOR, which constitutively inhibits autophagy in homeostasis. Signals such as starvation, energy depletion, absence of growth factors and amino acids, are transduced by AMPK or AKT kinases, leading to mTOR inhibition (29, 33). Consequently, ULK1 complex activation occurs, promoting Beclin1 interactome phosphorylation, which enables PI3K Vps34 to produce PI3P, relevant to phagophore isolation from intracellular membrane structures such as the endoplasmic reticulum (ER) (34, 35). In parallel, ATG9 and VMP1 are responsible for lipid recruitment to the isolated membrane, participating in its biogenesis (29). Afterward, phagophore elongation and maturation occurs, which depends on two ubiquitin-like conjugation systems: 1) the oligomeric ATG12-ATG5-ATG16 complex and 2) LC3 (LC3I), after being conjugated with a phosphatidylethanolimine (LC3II), which together promote phagophore elongation and membrane closure to form the autophagosome (29, 35). Selective autophagy occurs in response to misfolded proteins and damaged organelles, which are targeted for degradation by sequestosome-like proteins such as SQSTM1/p62 and BNIP3. These proteins, which have LC3-interacting regions, recognize ubiquitinated proteins and damaged mitochondria, for instance, taking them to the emerging autophagosome to be engulfed (29, 36).

Other kinases that also regulate starvation-induced autophagy, such as JNK1 and eIF2α, initiate autophagy in response to oxidative and ER stress, and hypoxia (**Figure 1B**). eIF2α transduces stress signals imposed by ER-misfolded proteins, reduced oxygen levels and increased levels of reactive oxygen species (ROS). Hypoxia and ROS can also induce autophagy *via* JNK1 (29). Additionally, hypoxia induces autophagy *via* HIF by weakening the Bcl2 inhibitory interaction with Beclin1 or by inducing BNIP3 to deliver mitochondria for autophagic degradation (29, 37). Abnormal membrane potential in damaged mitochondria (**Figure 1B**), which are potent autophagy inducers, can also be sensed by voltage-dependent kinases, inducing ubiquitination of mitochondrial proteins, which are subsequently targeted by SQSTM1/p62 and BNIP3, directing mitochondria to be degraded by autophagy (29, 38). Apoptosis (**Figure 1B**), which is often a consequence of mitochondrial damage, can be counteracted by autophagy, both occurring almost in a mutually exclusive manner. Upon dissociation from Bcl2, Beclin1 induces autophagy *via* PI3K Vps34 (34), while Bcl2 inhibits apoptosis by counter regulating mitochondrial membrane permeabilization (39, 40). This supports autophagy’s role as a cytoprotective mechanism that can circumvent cell death by abrogating manageable levels of cellular stress (41, 42).

Innate immunity (**Figure 1B**) also modulates autophagy, especially in the context of infections. Since infections have been linked to nutritional stress, it is plausible to hypothesize that evolution led to the development of cellular mechanisms shared between the immune system and autophagy (31). TLRs trigger autophagy *via* TRIF/MyD88 signaling, weakening the Bcl2-Beclin1 interaction, which normally represses autophagy (43). Likewise, NLRs were also shown to induce autophagy by activating ATG16L1 in response to bacterial infections (44). Immune system activation by pathogens frequently culminates in cytokine production and inflammation. As such, autophagy can be activated to 1) attenuate inflammation by controlling immune system activation or 2) directly destroying pathogens (30, 31, 45). Th1 proinflammatory cytokines, such as TNF-α, are produced during infections and contribute to autophagy induction by mechanisms that are still poorly understood (46). TNF-α was shown to induce LC3 transcription and conversion into LC3II, Beclin1 synthesis and autophagosome accumulation in human muscle cells (47, 48). In addition, elimination of intracellular pathogens such as *T. gondii*, *Shigella* spp., and *Listeria* spp. by autophagy can also be tuned by TNF-α, supporting the role of proinflammatory cytokines in autophagy-dependent pathogen clearance (49, 50). On the other hand, Th2 cytokines, such as IL-10, are known to inhibit autophagy (46). IL-10 activation of its specific receptor was shown to inhibit LPS- (51) and starvation-induced autophagy in a PI3K- and AKT-dependent process (52).

Inflammation often promotes tissue damage while attempting to clear infections. Therefore, autophagy can be cytoprotective by degrading deleterious stimuli and controlling inflammation (**Figure 1B**). Autophagy-inflammasome interplay might represent the best example of how autophagy modulates inflammation (53, 54). Autophagy is known to negatively regulate inflammasomes and IL-1β production (54). NLRP3 and IL-1β levels were shown to be increased in ATG16L1 knockout mice challenged with bacterial toxins, suggesting a negative regulation of the NLRP3 inflammasome by autophagy (55). This probably occurs due to direct targeting of inflammasome-associated proteins for degradation. AIM2, ASC, Caspase-1, and IL-1β were shown to increase during autophagy inhibition, which was shown to be dependent on ubiquitination and SQSTM1/p62 selection (56, 57). Alternatively, autophagy can target pathogens for degradation (**Figure 1B**). It was shown that *Sindbis* and *Chikungunya* viral proteins (58, 59) and bacteria such as *L. monocytogenes* and *S. typhimurium* (60, 61) are selected by SQSTM1/p62 for autophagic degradation. To a similar extent, intracellular parasites such as *Plasmodium* spp. and *T. gondii* are also targeted by autophagy (62). However, unlike conventional autophagy, proteins such as LC3 may directly coat the parasitophorous vacuole instead of directing the parasite to be enwrapped by autophagosomes in a process known as LC3-associated phagocytosis (62).

Autophagy has an important role in maintaining tissue homeostasis in response to nutritional, oxidative and immunological stress. Likewise, gestation demands significant levels of placental plasticity and adaptation to ensure fetal development (63). Therefore, placental autophagy is likely to be fundamental to a healthy pregnancy, maintaining local homeostasis in response to metabolic and immunological stressors with the potential to jeopardize healthy gestational outcomes.

### Placental Autophagy in Healthy and Complicated Pregnancies

The role of placental autophagy during gestation is unclear. However, it is believed that placental autophagy functions as a survival mechanism while maintaining local homeostasis (64), opposing the misleading definition that classifies autophagy as a cell death mechanism (41). Embryonic development was reported to be autophagy dependent, as genetic ablation of ATG5 (65), Ambra1 (66), and Beclin1 (67) promotes embryonic lethality by impairing germinal and embryonic development. Knocking-out genes, such as *Atg3*, *Atg9*, *Atg16l1* and *Atg7*, failed to induce embryonic lethality but promoted neonatal death after birth (28). Despite current knowledge on this topic, the mechanisms leading to poor outcomes in autophagy-deficient pregnant mice are still unclear.

Logically, one could expect autophagic abnormalities in complicated pregnancies. In uncomplicated pregnancies, placental levels of Beclin1 and LC3 should not vary significantly throughout gestation (68). However, the same is not true during pregnancy-related disorders. Preeclamptic women, for instance, who often experience hypertension, placental insufficiency, fetal growth restrictions and preterm delivery (69), frequently exhibit placental autophagy dysregulation. Increased LC3II and Beclin1 levels and diminished SQSTM1/p62 levels were found in placentas from preeclamptic women with fetal growth restrictions, suggesting overinduction of placental autophagy (70–73). Accordingly, autophagy was hypothesized to be etiologically linked to these outcomes during preeclampsia (71, 73, 74). However, pregnant mice knockout for *Atg7* and *Atg9a* present preeclamptic-like symptoms and evidence of fetal growth restrictions, suggesting that functional autophagy is necessary for healthy gestations (75–77). The reason that placental autophagy is induced above basal levels during complicated pregnancies remains a mystery. Evidence indicates that autophagy is activated in response to hypoxia, protein aggregation, and oxidative stress or to maintain local homeostasis in response to inflammation (74, 78).

Other studies have shown an apparent link between impaired autophagy and inflammation-induced preterm labor. Women experiencing preterm delivery due to infection-induced inflammation have impaired placental autophagy, characterized by reduced levels of ATG16L1 and LC3II and increased levels of SQSTM1/p62 (79). Likewise, downregulation of placental autophagy genes such as *Atg4c*, *Atg7*, and *LC3B* was also reported in a murine model of inflammation-induced preterm delivery (80). Since delivery itself (either preterm or at term) relies on mechanisms of inflammation, such as the secretion of proinflammatory cytokines such as IL-1β (81, 82), its exacerbation frequently results in labor anticipation; therefore, counterregulatory mechanisms are necessary to ensure placental homeostasis. Since autophagy was shown to downregulate inflammasomes, for instance (54–57), it is plausible to hypothesize that it might participate in the regulation of placental inflammation. In fact, one study reported diminished levels of Beclin1, ATG3, ATG5, ATG12 and ATG16L1 in placental tissue after spontaneous delivery. *Ex vivo* stimulation of this tissue with LPS led to increased liberation of IL-1β that was augmented by autophagy inhibition and attenuated during autophagy induction, suggesting that this homeostatic process downmodulates placental inflammation in preterm deliveries (83).

Therefore, placental autophagy might have a cytoprotective role against stress induced during pregnancy (64). In addition, studies have provided evidence of a link between infection-induced inflammation during pregnancy and placental autophagy (79, 80), which might be critical for dysregulating placental homeostasis, contributing to poor outcomes during pregnancy-related diseases, such as MiP.

## Is There a Role for Placental Autophagy in MiP?

### Evidence of Placental Autophagy Dysregulation in Pregnant Women With Malaria

Low birth weight in MiP is known to have a multifactorial etiology. A combination of impaired placental perfusion, dysfunctional endocrine signaling and transplacental nutrient transportation seems to be the most significant cause (3). However, a link between placental autophagy and MiP pathogenesis was established, especially in the context of local inflammation. Placental intervillositis (monocyte infiltrate in the tissue) during MiP was shown to be associated with low birth weight and reduced IGF levels, a hormone with significant importance to fetal growth and transplacental nutrient transportation (3, 84). Accordingly, it was observed that placental inflammation characterized by the presence of cytokines such as IL-1β and intervillositis was also associated with decreased expression of amino acid and glucose transporters, and low birth weight (85, 86). It was hypothesized that reduced transplacental nutrient uptake due to decreased expression of nutrient transporters was controlled by mTOR as a consequence of diminished levels of circulating IGF and local inflammation (63, 87). Kinases rpS6, 4E-BP1, and AKT (mTOR targets) phosphorylation levels were diminished in placentas from women with MiP-associated intervillositis and constitutive mTOR activation in trophoblasts was shown to upregulate amino acid transporter activity even when incubated with conditioned culture medium from monocytes co-cultured with *P. falciparum*-infected erythrocytes, proving the existing link between mTOR activity, downregulation of transplacental amino acid transport and MiP-associated intervillositis (88). Although autophagy activation is directly associated with mTOR inhibition in the absence of nutrients and growth factors (29), no hypotheses were raised at that time. Afterward, evidence of placental autophagy dysregulation in *P. falciparum*-infected women was presented. Placental parasitemia and intervillositis were associated with increased LC3II protein levels and autophagosomes/lysosomes in the placenta (induced autophagy); however, reduced Rab7 and unaltered SQSTM1/p62 levels supported autophagy blockage with consequent impaired degradation of autophagosome content (89). Likewise, our findings suggested abnormal autophagy regulation in placentas from *P. falciparum*-infected women, characterized by reduced *ULK1*, *BECN1* and *MAP1LC3B* mRNA levels and reduced ULK1, Beclin1 and LC3II protein levels. These differences were more prominent in placentas diagnosed with PM, which experience higher levels of local inflammation (90).

Despite evidence supporting the existence of impaired placental autophagy during MiP, its role in disease pathogenesis remains unclear. Dissecting placental autophagy’s role during MiP is extremely important to understand whether this mechanism has a pathogenic or a cytoprotective function that can be exploited to improve MiP prognosis.

### Hypothetical Modulators of Placental Autophagy During MiP

**Figure 2:**
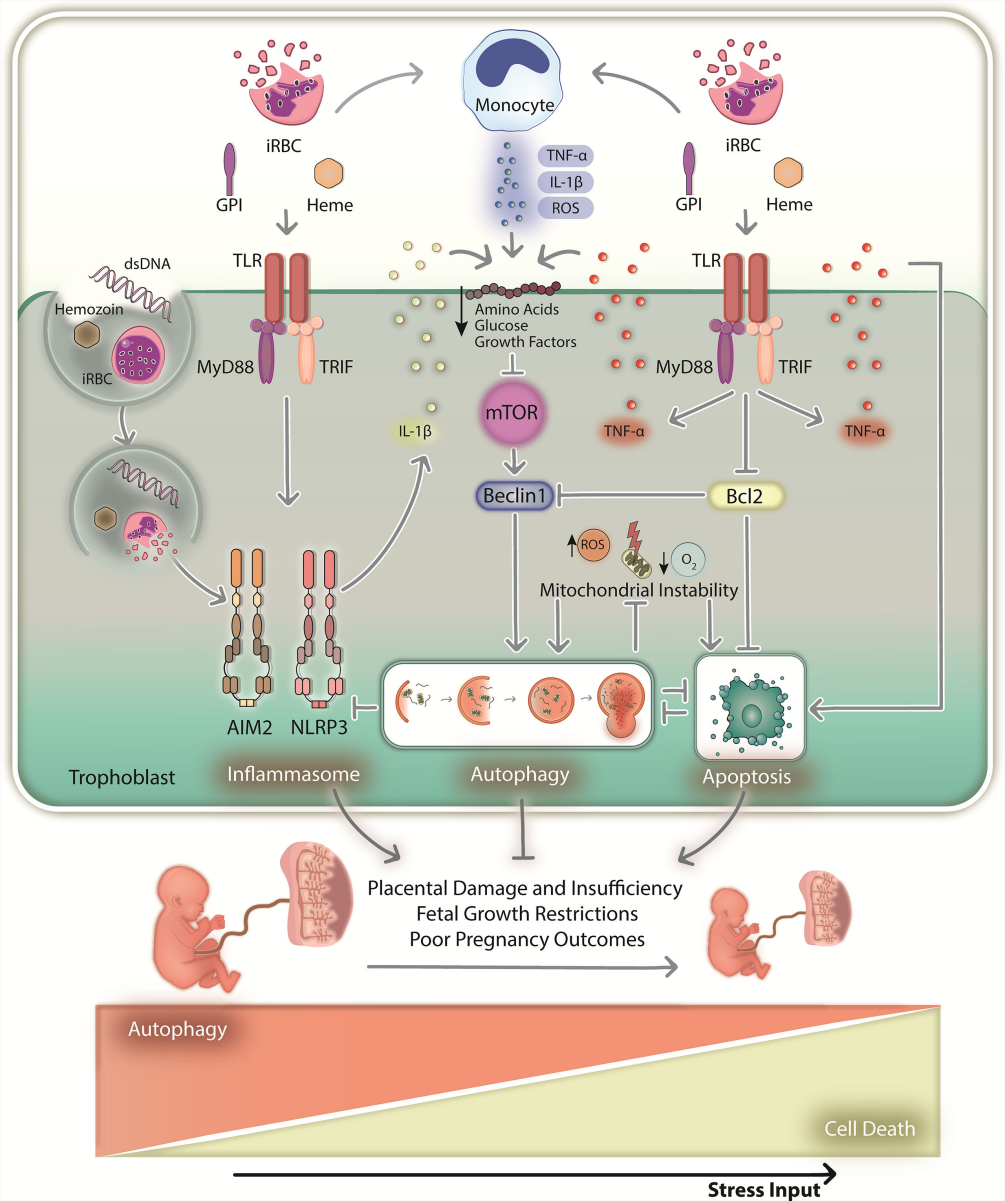
The hypothetical role of placental autophagy during malaria in pregnancy. During MiP, immune system activation in response to local infection results in inflammation and placental homeostasis imbalance. Monocytes and trophoblasts recognize infected red blood cells and parasite-derived antigens (dsDNA, GPI and hemozoin) *via* TLRs, which promote the downstream production of cytokines such as TNF-α and IL-1β (upon being processed by the inflammasome). TLR-MyD88-TRIF signaling might trigger autophagy by disrupting the Bcl2-Beclin1 interaction or through the paracrine action of the produced cytokines. In addition, inflammation leads to the downregulation of glucose/amino acid transporters, consequently activating autophagy upon mTOR inhibition. The hypoxic environment and ROS produced by monocytes might also induce autophagy by promoting oxidative stress and mitochondrial instability. Consequent activation of apoptotic machinery might also activate autophagy. As such, a multitude of stimuli might induce placental autophagy above basal levels, which might function as a cytoprotective mechanism that maintains local homeostasis and proper gestational development when stress intensity is low. In these circumstances, autophagy might circumvent lethal outcomes that may arise from activation of cell death mechanisms. However, persisting infection and sustained inflammation might increase cellular stress by activating programmed cell death mechanisms that regulate pyroptosis and apoptosis, for instance. First, autophagy might act as a survival mechanism by promoting nutrient recycling and destroying death signals such as inflammasome machinery and damaged mitochondria. However, if stress input increases above a given threshold, cell death executors such as apoptotic caspases might degrade autophagy-related proteins, consequently impairing their cytoprotective functions. Therefore, tissue homeostasis will not be maintained, resulting in the dysregulation of placental physiology, eventually leading to MiP poor pregnancy outcomes. dsDNA, parasite double-stranded DNA; iRBC, infected red blood cells; GPI, glycosylphosphatidylinositol; ROS, reactive oxygen species; TLR – Toll-like receptor.

One can consider that parasite virulence factors such as VAR2CSA and microvesicles might have a role on placental autophagy modulation. Pregnant women diagnosed with PM (VAR2CSA-dependent sequestration of infected erythrocytes) experience a significant modulation of autophagy when compared to women without PM (90). Additionally, infected erythrocytes selected for binding trophoblasts activate JNK1 in these cells (12), which is a known autophagy inducer (29). However, experiments with null-VAR2CSA parasites were missing so we cannot rule out the hypothesis that observed effects might still occur in a binding independent manner and simply due to parasite antigen recognition. *Plasmodium*-derived microvesicles, which carry parasite-derived antigens and activate the immune system, are transferred to astrocytes by LC3-associated phagocytosis, with implications to neuroinflammation and cerebral malaria pathogenesis (91). Besides, microvesicles can also trigger inflammation by activating TLR4-MyD88 axis (92), which is linked to autophagy modulation as previously discussed. Although we cannot discard the hypothesis that some of these virulence factors regulate autophagy, it seems that placental autophagy during MiP is mostly modulated by immune signals and inflammation with possible implications to disease pathogenesis (**Figure 2**).

During MiP, trophoblasts produce chemokines upon parasite recognition, recruiting monocyte-producing cytokines and ROS to clear local infection. However, excessive inflammation downregulates IGF signaling and glucose/amino acid transportation, activating autophagy upon mTOR inhibition (85, 86, 88, 89). Autophagy might also be triggered upon parasite sensing by TLRs (92), which induce autophagy through the MyD88-TRIF interaction with the Bcl2-Beclin1 complex (43). In fact, TLR-MyD88 signaling, which initiates inflammation by promoting the production of cytokines such as TNF-α, and IL-1β (by inducing its transcription and posttranslational maturation through the inflammasomes), is implicated in the outcomes of MiP, since genetic depletion of TLR4 and MyD88 improves pregnancy outcomes in an experimental murine model of MiP (93–96). In parallel, MiP-associated hypoxia and ROS (3, 27) can promote mitochondrial instability, increasing oxidative stress and cell death *via* apoptotic pathways. Placental apoptosis occurs in pregnant mice and women with MiP, characterized by increased trophoblast apoptosis, active Caspase-3, and reduced Bcl2 levels (97, 98). Accordingly, reduced Bcl2 levels and damaged mitochondria might also activate autophagy *via* a canonical pathway dependent on Beclin1. Inflammasomes, which potentiate inflammation and cell death *via* pyroptosis in response to parasite antigens and danger signals, can also induce autophagy. Placental NLRP3- and AIM2-dependent IL-1 axis activation is associated with MiP outcomes (99), which in principle can be downregulated by autophagy if overactivated (54). In addition, autophagy can be induced by proinflammatory cytokines such as TNF-α, which induce apoptosis *via* death receptors and are associated with MiP poor outcomes (14, 19, 20).

### A Hypothetical Role for Placental Autophagy During MiP

If autophagy promotes survival in response to stress, if it is essential for gestational development, and is downregulated in pregnancies complicated by infection-induced inflammation such as MiP, one can hypothesize that poor outcomes will result from placental homeostasis imbalance, which cannot be maintained by autophagy due to nonmanageable stress levels caused by infection. A cytoprotective role for placental autophagy during MiP can be hypothesized when stress levels are not lethal and cell death mediated by apoptotic mechanisms, for instance, is not triggered in response to stress (**Figure 2**). Autophagy can counteract cell death *via* apoptosis, for instance, by degrading damaged mitochondria and proapoptotic cytosolic signals; however, stress levels will eventually exceed a given threshold, and autophagy will not be able to circumvent the lethal outcome. The interplay between autophagy and apoptosis is mostly of an inhibitory nature (42). Therefore, proapoptotic caspases may eventually cleave regulatory proteins, downmodulating autophagy and possibly aborting its cytoprotective function, resulting in total dysregulation of cellular homeostasis and ultimately, cell death. This sequence of events may promote placental damage and dysregulation of local homeostasis, ultimately contributing to MiP poor outcomes.

In this context, we hypothesize that placental autophagy plays a cytoprotective role during MiP by promoting nutrient recycling and controlling inflammation and cellular damage. However, this activity cannot be properly exerted due to the nonmanageable levels of inflammation, cell death and tissue damage observed in placentas from women infected with *P. falciparum*, contributing to impaired gestational development and poor pregnancy outcomes.

## Data Availability Statement

The original contributions presented in the study are included in the article. Further inquiries can be directed to the corresponding author.

## Author Contributions

AB drafted the manuscript, collected information from the literature and drafted the figures. AJ drafted the manuscript. CM and SE supervised and reviewed the manuscript. All authors contributed to the article and approved the submitted version.

## Funding

This manuscript was primarily funded by the São Paulo Research Foundation – FAPESP (2018/20468-0 and 2020/06747-4 to CM, 2020/03163-1 to SE) and National Council for Scientific and Technological Development – CNPq (408636/2018-1 and 302917/2019-5 to CM, 304033/2021-9 to SE). AB and AJ were supported by FAPESP fellowships (2017/03939-7 and 2019/12078-0, respectively).

## Conflict of Interest

The authors declare that the research was conducted in the absence of any commercial or financial relationships that could be construed as a potential conflict of interest.

## Publisher’s Note

All claims expressed in this article are solely those of the authors and do not necessarily represent those of their affiliated organizations, or those of the publisher, the editors and the reviewers. Any product that may be evaluated in this article, or claim that may be made by its manufacturer, is not guaranteed or endorsed by the publisher.
